# Lifetime occupational physical demand and risk of hip fracture in older adults: A retrospective cohort study

**DOI:** 10.1097/MD.0000000000047105

**Published:** 2026-01-09

**Authors:** Lei Wang, Qiang Xu, Qinqing Xie, Jie Zhang, Xiongxiong Wang, Huatuo Cao

**Affiliations:** a Department of Orthopedics, Affiliated Hospital of Yan’an University, Yan’an, Shaanxi Province, China; b Department of Orthopedics, The People’s Hospital of Yan’an, Yan’an, Shaanxi Province, China.

**Keywords:** hip fracture, observational study, occupational physical demand, older adults

## Abstract

Hip fractures in the elderly, especially among the Chinese population, are among the most serious complications of osteoporosis. As age increases, the incidence of these fractures rises. In this study, we seek to identify a range of lifetime occupational physical-demand factors that may serve as potential indicators of hip-fracture risk in Chinese individuals aged 65 years and older. Our aim is to inform preventive strategies and occupational health policies by enhancing understanding of how long-term physical work demands contribute to fracture risk across the aging population. This study employed a retrospective cohort design and was conducted at the Affiliated Hospital of Yan’an University. We included patients who underwent surgical treatment for intertrochanteric fractures or femoral neck fractures between January 2017 and December 2021. The study followed the STROBE (Strengthening the Reporting of Observational Studies in Epidemiology) guidelines to enhance transparency and methodological reporting. A total of 434 patients were included in this study, comprising 95 with light, 297 with moderate, and 42 with heavy occupational physical demand. Unadjusted multivariate logistic analysis revealed that moderate occupational physical demand was associated with a decreased risk of hip fracture (OR = 3.57, 95% CI: 2.01–6.33, *P* <.0001; compared to heavy occupational physical demand). Adjusted multivariate logistic model Ⅰ showed that moderate occupational physical demand (OR = 3.35, 95% CI: 1.75–6.44. *P* = .0003; compared to heavy occupational physical demand) remained associated with hip fracture. Fully adjusted multivariate logistic model Ⅱ also showed that moderate occupational physical demand (OR = 2.83, 95% CI: 1.51–5.97, *P* = .0032; compared to heavy occupational physical demand) remained associated with hip fracture. Compared to heavy physical demand, moderate occupational physical demand might be associated with a higher risk of hip fracture among older adults.

## 1. Introduction

Hip fracture remains a significant public health challenge, especially among the elderly with osteoporosis, due to its strong association with increased morbidity, disability, and mortality. The incidence of hip fractures rises sharply with age, and currently, an estimated 14 million individuals worldwide experience a hip fracture each year.^[1]^ Despite improvements in surgical techniques and healthcare, mortality persists, while the overall incidence continues to increase due to aging populations and a broad array of risk factors.^[2]^ Postoperative recovery in the elderly is often lengthy and complicated, with adverse events including pneumonia, urinary tract infections, and venous thromboembolism. Factors such as advanced age, male sex, and delayed surgical intervention (>48 hours after admission^[3]^) contribute to higher mortality, while nutritional and metabolic factors, particularly BMI, influence postoperative outcomes. Obesity is linked to higher risks of complications, whereas being underweight is associated with higher perioperative transfusion rates.^[4,5]^ However, evidence on the BMI-mortality relationship in hip fracture contexts remains limited.^[6]^

Beyond intrinsic factors, lifelong occupational physical demands may influence bone health and fracture risk.^[7,8]^ Prolonged exposure to heavy lifting, repetitive movements, or standing can affect bone mineral density and musculoskeletal resilience, with mixed findings in the literature. Some studies suggest no significant association, while others indicate increased or decreased risk with cumulative occupational exposure.^[9,10]^ Given the substantial societal burden and the inconsistent evidence, this study aims to elucidate the relationship between lifetime occupational physical demand and hip fracture risk in the elderly, with potential implications for preventive strategies and occupational health policy.

## 2. Materials and methods

### 2.1. Study design and population

This is a retrospective, observational cohort study conducted at a tertiary-care academic hospital. We used electronic medical records to identify all eligible patients who underwent surgical treatment for hip fractures, specifically intertrochanteric or femoral neck fractures, from January 1, 2017 to December 31, 2021. Data collection for exposure, outcomes, and covariates occurred within the same period. The study adhered to STROBE guidelines for reporting observational studies. The study protocol was approved by the institutional review board/ethics committee of the participating institution, with a waiver of informed consent granted for this retrospective chart review.

### 2.2. Eligibility criteria

#### 2.2.1. Inclusion criteria

Age ≥ 65 years at the time of fracture.

Sustained an intertrochanteric or femoral neck fracture between January 2017 and December 2021.

Underwent surgical management for the fracture at the study institution.

#### 2.2.2. Exclusion criteria

History of systemic corticosteroid use >3 months.

Preexisting rheumatoid disease.

Malignancy or cancer-related conditions.

Thyroid or parathyroid diseases.

Prior history of hip fractures or other high-energy hip traumas.

Incomplete medical records preventing assessment of key variables or outcomes.

Rationale for study population size.

The study size comprises all eligible consecutive patients within the specified window who had sufficient data for prespecified analyses. No formal a priori sample size calculation was performed because the objective was to describe associations in a real-world retrospective cohort using all eligible cases. The final analytic sample was 434 after excluding 21 patients for the reasons above or due to missing essential data.

### 2.3. Data collection

**Figure 1.**
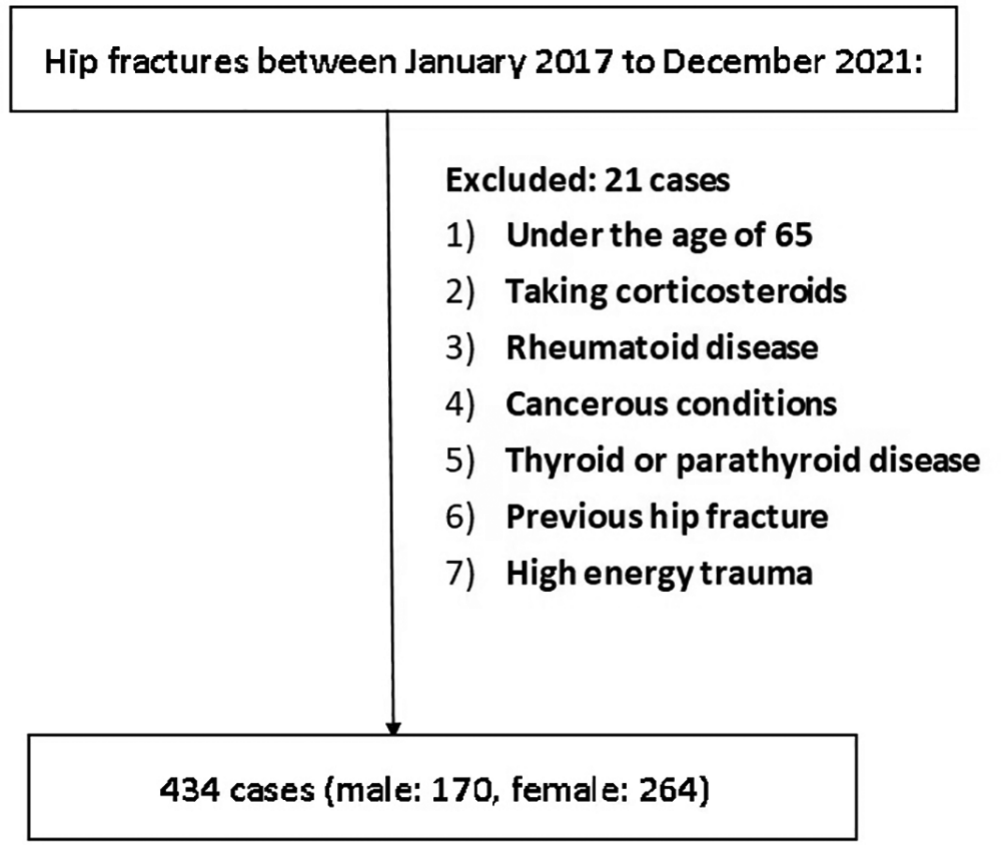
A flowchart illustrating patients’ enrollment process.

Data were collected by 3 independent researchers trained in standardized data abstraction. In the event of discordant entries, discrepancies were resolved by discussion and consensus; when necessary, a 4th reviewer adjudicated. Where information was not available in the medical records, data were treated as missing and analyzed using complete-case analysis for the primary analyses. A flow chart (Fig. 1) will depict screening, exclusions, and the final analytic sample.

### 2.4. Variables and assessment methods

Demographics: age, sex, ethnicity, residential address.

Anthropometrics: height, weight, body mass index (BMI).

Comorbidities: diabetes mellitus, hypertension, and other chronic conditions as documented.

Fracture characteristics: fracture type (intertrochanteric vs femoral neck), mechanism of injury (where available), season of admission.

Socioeconomic status and occupation: job title coded according to the Australian Classification of Standard Occupation (ASCO).

Occupational physical demand: classified into 3 categories – light, moderate, and heavy (Table 1) – based on a framework described by L. K. P. Suen,^[10]^ using routine occupational duties and energy expenditure estimates. In the event of discordant entries, discrepancies were resolved by discussion and consensus; when necessary, a 4th reviewer adjudicated.

**Table 1:** Classification of occupational physical demand levels.

Occupational physical demand level	Description	Example of occupations
Light	Standing, sitting, walking, little or no lifting	Secretary, office, clerk, Sales clerk, driver
Moderate	Usually lifting, carrying light loads, climbing stairs	Factory worker (packers), domestic helper, nurse, restaurant worker, teacher
Heavy	Heavy work, carrying heavy loads	Farmer, heavy-duty factory worker

Additional variables: length of stay, admission season, and available laboratory/imaging findings used for clinical context.

Data quality and bias mitigation.

Selection bias was minimized by including all consecutive eligible patients within the study period.

Information bias was mitigated by independent data abstraction with predefined coding rules; discrepancies were resolved by consensus.

Confounding was addressed by collecting relevant covariates (age, sex, BMI, comorbidities) to enable multivariable adjustment in prespecified analyses.

Temporal bias was minimized by using a fixed study window and standardized data collection across years.

## 3. Statistical analysis

EmpowerStats (X&Y Solutions, Boston) and R 3.4.3 software were used for statistical analysis. Continuous data were presented as mean ± standard deviation, while categorical variables were presented as percentages.

Three multivariate logistic regression models were employed to analyze the relationship between occupational physical demand and hip fracture. Model 1 (unadjusted) did not include any adjustments. Model 2 (Adjusted I) incorporated adjustments for age, sex (male, female), ethnicity (Han, Hui, Zang, Man, others), weight, scene (home, road, work, sports, others, stairs), residential address (city, country), diabetes (no, yes), hypertension (no, yes), height, and reason (FPA, VP, EM, CY, MEF, CRI, OSAP, OUR). Model 3 (Adjusted II) included additional adjustments with smoothing for age, sex (male, female), ethnicity (Han, Hui, Zang, Man, others), weight (smooth), scene (home, road, work, sports, others, stairs), residential address (city, country), diabetes (no, yes), hypertension (no, yes), height (smooth), and reason (FPA, VP, EM, CY, MEF, CRI, OSAP, OUR). Two-sided *P* <.05 was considered statistically significant.

## 4. Ethics

The study protocol was approved by the Ethic Committee of Affiliated Hospital of Yan’an University (Approval No. S-S20240036), and conducted in accordance to local ethical guidelines. The requirement for informed consent was waived, as all data were de-identified to protect patient’s right to privacy.

## 5. Results

Among the initially recruited 455 patients, 21 were excluded due to pathological fractures caused by bone tumors (n = 9) and incomplete data (n = 12), resulting in a total of 434 patients include in this study (Fig. 1). Compared to patients with heavy occupational physical demand, those with moderate occupational physical demand were older (74.24 ± 6.32 vs 72.94 ± 6.78 years, *P* = .001), and showed a higher proportion of hip fractures (43.8% vs 17.9%, *P* <.001). Additionally, significant differences were observed in scene of fracture (*P* = .008), residential address (*P* <.001), and reason (*P* = .003) (Table 2).

**Table 2:** Basic and clinical characteristics.

	Heavy (n = 95)	Moderate (n = 297)	Light (n = 42)	*P*
Age	72.94 ± 6.78	74.24 ± 6.32	77.26 ± 6.27	.001
Weight (kg)	57.07 ± 6.68	56.58 ± 6.89	57.05 ± 6.70	.787
High (meter)	1.62 ± 0.07	1.62 ± 0.06	1.64 ± 0.07	.406
BMI (kg/m^2^)	21.63 ± 2.14	21.43 ± 1.99	21.28 ± 1.92	.578
Gender				
Male	42.1	37.4	45.2	.498
Female	57.9	62.6	54.8	
Ethnic				
Han	96.8	97.0	100.0	.870
Hui	2.1	1.3	0.0	
Zang	1.1	0.3	0.0	
Man	0.0	0.3	0.0	
Others	0.0	1.0	0.0	
Scene of fracture				
Home	40.0	63.3	61.9	.008
Road	49.5	28.3	28.6	
Work	4.2	0.7	2.4	
Sports	2.1	1.7	2.4	
Others	3.2	5.4	4.8	
Stairs	1.1	0.7	0.0	
Address				
City	65.3	80.1	95.2	<.001
Country	34.7	19.9	4.8	
Diabetes				
No	95.8	94.9	95.2	.946
Yes	4.2	5.1	4.8	
Hypertension				
No	82.1	87.2	83.3	.418
Yes	17.9	12.8	16.7	
Reason				
FPA	7.4	1.7	4.8	.003
VP	22.1	14.5	0.0	
EM	1.1	0.3	2.4	
CY	1.1	0.0	0.0	
MEF	1.1	0.0	0.0	
CRI	1.1	0.7	2.4	
OSAP	63.2	77.4	81.0	
OUR	3.2	5.4	9.5	
Season of admission				
Spring	26.3	21.9	23.8	.851
Summer	30.5	32.0	23.8	
Autumn	11.6	14.8	19.0	
Winter	31.6	31.3	33.3	
Hip fracture				
No	82.1	56.2	69.0	<.001
Yes	17.9	43.8	31.0	

BMI = body mass index, CD = cartilage degeneration, CRI = cortical remodeling instability, EM = excessive microtrauma, FPA = femoral proximal alignment, MEF = muscular endurance fatigue, OSAP = osteoporotic structural adaptation progression, OUR = osteoclast unregulated resorption, VP = vascular perfusion.

Approximately 9.68% of cases with light occupational physical demand were office-related. Within the moderate-activity occupations, accounting for 68.43% of cases, the majority were teachers (68.2%), with a small proportion in other occupations (0.23%). Conversely, the heavy-activity category exhibited less diversity, with 21.89% of cases being farmers (14.06%) and workers (7.83%).

Unadjusted multivariate analysis revealed that moderate occupational physical demand (OR = 3.57, 95% CI: 2.01–6.33, *P* <.0001; compared to heavy occupational physical demand) were significantly associated with an increased risk of hip fracture. Following adjustment for potential confounding factors, the impact of occupational physical demand on hip fracture risk was assessed based on 3 multivariate logistic regression analysis models (Table 3). In adjusted model I, moderate occupational physical demand was associated with a higher hip fracture risk (OR = 3.35; 95% CI: 1.75–6.44, *P* = .0003). In fully adjusted model II, moderate occupational physical demand remained associated with a higher hip fracture risk (OR = 2.83, 95% CI: 1.51–5.97, *P* = .0032).

**Table 3:** The multivariate logistics analysis.

Occupation physical demand	Nonadjusted		Adjusted I		Adjusted II	
	OR (95% CI)	*P*	OR (95% CI)	*P*	OR (95% CI)	*P*
Heavy	Ref		Ref		Ref	
Moderate	3.57 (2.01–6.33)	<.0001	3.35 (1.75–6.44)	.0003	2.83 (1.42–5.64)	.0032
Light	2.06 (0.89–4.76)	.0919	1.65 (0.65–4.18)	.2931	1.40 (0.52–3.79)	.5081

CI = confidence interval, OR = odds ratio.

## 6. Discussion

Along with the increased age of the global population, the incidence of hip fractures has steadily increased as well.^[11,12]^ While the fracture itself must be addressed, long-term hospital stays, expensive rehabilitation treatments, and additional complications have led to larger economic burdens on both the patient and provider^[2]^). Therefore, gaining a better understanding of the epidemiology and possible correlating demographical factors for osteoporotic fractures is helpful in addressing efforts to prevent or better treat such fractures in high-risk groups like the elderly.^[12]^

This retrospective cohort study among Chinese older adults examined the association between occupational physical demand and hip fracture risk. Occupational physical demand was categorized into 3 levels: light, moderate, and heavy.^[10]^ Previous research has emphasized the importance of occupational physical activity for skeletal health; notably, heavy physical demands are often considered beneficial for bone status^[13,14]^ and may reduce the risk of hip fractures.^[15,16]^ Consistent with these findings, Damilakis et al^[17]^ suggested that lifelong occupational physical activity positively influences bone health in postmenopausal farmers. Our results corroborate this, indicating that higher occupational physical demands, specifically at the heavy level, are associated with a decreased risk of hip fracture.

However, our findings also reveal that individuals engaged in moderate levels of occupational physical demand – particularly those working as teachers, helpers, or nurses – exhibited an increased risk of hip fracture. In contrast, no significant association was observed among those with light occupational demands. These results partially contradict certain studies from Western populations, such as the UK study,^[18]^ which reported that sedentary occupations at age 50 were associated with a higher risk of subsequent hip fractures. This discrepancy may stem from differences in occupational environments, lifestyle factors, or healthcare systems between populations. In China, those engaged in light physical work often have a relatively good educational background and a high socioeconomic status (SES). Regarding (SES), our findings and prior literature suggest a protective effect of higher SES against hip fracture risk.^[19]^ This may be attributed to better nutrition, healthier lifestyle behaviors – such as lower smoking and alcohol consumption – and higher access to medical care, which collectively contribute to higher bone mineral density and fewer environmental hazards at home. Experimental studies have consistently demonstrated that poor dietary calcium intake,^[20,21]^ heavy alcohol consumption,^[22]^ and cigarette smoking^[23]^ are associated with increased fracture risk. Higher SES individuals may also benefit from safer living environments and superior fall preventative measures.

The observed increased risk of hip fracture among those engaged in moderate occupational physical activities might be attributable to the nature of such work, which could involve irregular or strenuous activities that predispose individuals to falls or injuries, especially in older age. Alternatively, it is possible that moderate occupational demands do not provide sufficient mechanical stimulus for optimal bone strengthening, contrasting with the protective effects observed in heavier occupational activity. Furthermore, differences in occupational environments, lifestyle factors, SES, and healthcare systems likely explain the observed gaps in hip fracture risk between our Chinese cohort and Western cohorts. So, it is suggested to carry out additional in-depth research to achieve more reliable findings.

In summary, while heavy occupational physical demands appear to confer protective effects on bone health and reduce hip fracture risk, moderate-demand occupations may paradoxically increase fracture risk among the elderly. These findings underscore the complexity of occupational activity’s influence on skeletal health and highlight the need for further prospective, in-depth research to validate these associations and elucidate underlying mechanisms.

Despite predefined criteria, residual heterogeneity may remain due to unmeasured factors (e.g., smoking, alcohol use, nutrition, osteoporosis treatment, BMI trajectory, perioperative care variability) and potential exposure misclassification inherent to retrospective occupational history. The generalizability of findings may be limited to older adults with hip fractures treated in similar hospital settings. Further prospective studies or population-based registries with standardized exposure assessment and comprehensive covariate capture are warranted to validate these associations and to quantify dose–response relationships between lifetime occupational physical demand and hip fracture risk.

In conclusion, this retrospective observational study revealed an association between occupational physical demand and hip fracture risk among Chinese older adults. Notably, when the heavy occupational physical demand served as the reference, it was found that the moderate occupational physical demand was correlated with an increased risk of hip fracture, whereas the light occupational physical demand exhibited no significant difference. Further large-scale prospective research is warranted to enhance the robustness of the findings and promote targeted preventive measures.

## Author contributions

**Conceptualization:** Lei Wang, Qiang Xu, Qinqing Xie, Jie Zhang, Xiongxiong Wang, Huatuo Cao.

**Data curation:** Lei Wang, Qiang Xu, Qinqing Xie, Jie Zhang, Xiongxiong Wang, Huatuo Cao.

**Formal analysis:** Lei Wang, Qiang Xu, Qinqing Xie, Jie Zhang, Xiongxiong Wang, Huatuo Cao.

**Investigation:** Lei Wang, Qiang Xu, Qinqing Xie, Jie Zhang, Xiongxiong Wang, Huatuo Cao.

**Methodology:** Lei Wang, Qiang Xu, Qinqing Xie, Jie Zhang, Xiongxiong Wang, Huatuo Cao.

**Writing – original draft:** Lei Wang, Qiang Xu, Qinqing Xie, Jie Zhang, Xiongxiong Wang, Huatuo Cao.

**Writing – review & editing:** Lei Wang, Qiang Xu, Qinqing Xie, Jie Zhang, Xiongxiong Wang, Huatuo Cao.
